# Anti-Cancerous Potential of Polyphenol-Loaded Polymeric Nanotherapeutics

**DOI:** 10.3390/molecules23112787

**Published:** 2018-10-27

**Authors:** Umeorah Ernest, Hai-Yan Chen, Ming-Jun Xu, Yasamin Davatgaran Taghipour, Muhammad Hassham Hassan Bin Asad, Roja Rahimi, Ghulam Murtaza

**Affiliations:** 1 Department of Biomedical Engineering, School of Engineering, China Pharmaceutical University, Nanjing 210009, China; isporunn@gmail.com (U.E.); mingjun_xu@stu.cpu.edu.cn (M.-J.X.); 2 Department of Medical Nanotechnology, School of Advanced Medical Sciences, Tabriz University of Medical Sciences, Tabriz 1416663547, Iran; y.davatgaran@gmail.com; 3 Department of Pharmacy, COMSATS University Islamabad, Abbottabad Campus 22060, Pakistan; hasshamasad@ciit.net.pk; 4 Department of Traditional Pharmacy, School of Traditional Medicine, Tehran University of Medical Sciences, Tehran 5165665931, Iran; rojarahimi@gmail.com; 5 Department of Pharmacy, COMSATS University Islamabad, Lahore Campus 54600, Pakistan; gmdogar356@gmail.com

**Keywords:** cancer therapeutics, polymeric carriers, nanoparticles, polyphenols, cell lines

## Abstract

Recent evidence has extensively demonstrated the anticancer potential of nutraceuticals, including plant polyphenols. Polymeric nanocarrier systems have played an important role in improving the physicochemical and pharmacological properties of polyphenols, thus ameliorating their therapeutic effectiveness. This article summarizes the benefits and shortcomings of various polymeric systems developed for the delivery of polyphenols in cancer therapy and reveals some ideas for future work.

## 1. Introduction

A large number of food ingredients have beneficial effects on human health. In particular, during the last decade, polyphenols and antioxidants have been extensively investigated for their therapeutic effectiveness after their intravenous administration [1].

Polyphenols contain a minimum of one aromatic ring, as well as hydroxyl groups ranging from a minimum of one ring. They are different from each other on the basis of the number of aromatic rings and phenol groups [2] and can be grouped into two main classes: flavonoids and non-flavonoids [3]. The former contains 15 carbon atoms, comprising two aromatic rings connected by a three-carbon link, while the latter contains heterogeneous compounds with phenolic acids having between one and six carbon atoms. Quercetin, kaempferol, apigenin, and myricetin belong to flavonoid class, while resveratrol, vanillin, and ellagitannins are examples of non-flavonoids.

Polyphenols are biologically active compounds, having useful effects against various chronic diseases, including cancer [4]. The biological activities of polyphenols are generally attributed to their antioxidant potential [5]. However, a comprehensive explanation for the biological effects of polyphenols is still uncertain [6]. In addition, their effects are also believed to be modulated via distinct actions on the signaling pathways at a cellular level [7].

Clinical studies on cancer therapy have reported a significant decrease in the therapeutic effectiveness of conventional cytotoxic compounds. The reduced efficacy is not only attributed to their unsuitable physicochemical properties, such as lipophilicity, but also to inappropriate pharmacokinetic features, including multidrug resistance, poor penetration into tumor microenvironment, and toxicity to non-diseased cells [8,9,10]. A wide array of research activities has been conducted to decipher these issues by several approaches, including the investigation of alternative anticancer compounds, as well as the development of targeted nanotherapeutics.

## 2. Polyphenol-Loaded Polymeric Nanotherapeutics for Cancer Treatment

The pathophysiology of cancer involves molecular-level changes in biological processes. Thus, in recent years, approaches have emerged to develop nanodiagnostic and nanotherapeutic modalities, such as lipid nanoparticles, nanohybrids, and polymeric nanoparticles [11,12,13]. In preclinical and initial clinical trials, these nanocarriers have exhibited excellent performance as drug delivery vehicles [14,15,16]. Nano-sized drug delivery systems have several promising features, including improved stability, enhanced solubility, and increased surface area to volume ratio. In addition, the surface properties of such carriers can be modified to attain controllable pharmacological and physicochemical features, thereby reducing barriers to effective chemotherapy in cancer [17]. Additionally, an ameliorated therapeutic index and diminished toxicity to healthy cells are also achieved through the nanotherapeutic approach [17]. It is remarkable that active and passive targeting could be used to deliver drugs to specific sites. These properties are significantly important for typical biologically active compounds, such as polyphenols for their translation into useful therapeutic modalities. Regardless of the promising progress in basic cancer biology at the preclinical level, polyphenols have inappropriate pharmacological properties, such as low bioavailability due to inefficient systemic access, and thus require high doses for optimum therapeutic effect [18]. Although in vitro studies have proved the biological effectiveness of polyphenols, these findings could not be achieved in vivo due to their instability in the physiological conditions of temperature, pH, and enzyme system. Their stability and therapeutic effectiveness could be improved by developing polyphenol-loaded nanotherapeutics. Therefore, biologically active polyphenols could be combined with nano-sized carriers to overcome the drawbacks of conventional anticancer therapy and develop a clinically efficacious treatment for cancer.

### 2.1. Polymer-Based Nanovesicles

Polymeric vesicles are prepared using amphiphilic block copolymers, which contain a lipophilic and a hydrophilic segment. These self-assembled structures have variable shapes and sizes, such as polymersomes [19] and micelles [20]. These vesicular systems have drug delivery capabilities and offer specific benefits.

Polymersomes are bilayered vesicles, composed of high molecular weight amphiphiles. Thus, they allow slow permeability of drugs due to the strong mechanical properties of their membranes [21]. Additionally, the surface of polymersomes is modified by using shell-producing, water-soluble, flexible polymers to reduce polymersomes–macrophages interactions [22]. Moreover, polymeric micelles are composed of a lipophilic core and a hydrophilic shell, into which lipophilic and hydrophilic drugs can be loaded and delivered, respectively [21]. These vesicles exhibit a narrow size distribution, ranging between 20 and 80 nm, and are long-lasting in systemic circulation [23,24,25], but, due to their poor stability, they undergo premature drug leakage in the bloodstream, resulting in reduced therapeutic efficacy and enhanced undesired effects. Polyphenol-loaded polymersomes and micelles have been synthesized by using natural polymers, such as dextran, chitosan, gelatin, casein, and polyethylene glycols PEG, due to their biodegradable and biocompatible features Table 1 and Table 2.

Gelatin–dextran micelles loaded with tea polyphenols were studied for their effect on breast cancer using MCF-7 cells and it was found that the encapsulated polyphenols had an enhanced efficacy compared with their free form [26]. Later on, this carrier was loaded with curcumin for the treatment of HeLa cancer cells. The results revealed an improvement in the pharmacokinetic and therapeutic properties of the encapsulated curcumin, compared with its control [27]. In addition, polyvinyl pyrrolidone–PEG conjugates were used to develop polymersomes loaded with *Cotinus coggygria* flavonoids for the treatment of glioblastoma [28]. Moreover, curcumin delivery systems were prepared by using protein-type polymers, such as gelatin, casein, and keratin [29,30,31,32,33]. In addition to their biocompatibility, these materials supported curcumin’s efficacy on cancerous cells of the lung and cervix [29,30]. Curcumin-loaded chitosan–stearic acid conjugates exhibited an improvement in the curcumin efficiency against colon cancer [34]. Curcumin polymersomes and micelles have been prepared with an aim of enhancing their anticancer activity. Owing to its stealth properties and biocompatible nature, PEG is extensively used in the fabrication of nanoparticulate systems. In vitro testing of PEG–polyanhydride esters and PEG–polylactic acid vehicles for curcumin and doxorubicin showed their synergism in HeLa and MCF-7 cancer cells. The polymer conjugates were prepared by a solvent evaporation technique [35,36]. The solvent evaporation-induced synthesis of curcumin-loaded micelles of polycaprolactone and PEG was aimed at the treatment of various cancers, such as breast [37] and ovarian [38] cancer cells in vitro, and colon [39], breast [40], and lung [41] in xenograft mouse models. The anticancer efficacy of these polycaprolactone–PEG–curcumin nanomicelles against lung and brain tumors was further enhanced through their modification by using different fatty acids, such as oleic acid, linoleic acid, and palmitic acid [42,43]. In some other studies, 1,2-distearoyl-*sn*-glycero-3-phosphoethanolamine-*N*-[methoxypolyethylene glycol-2000] was employed for the synthesis of curcumin micelles to treat colon and ovarian cancers in vitro and in vivo, showing synergism with doxorubicin [44,45] and paclitaxel [46]. These in vitro and in vivo studies depict the promising characteristics of the polymeric polymersomes and micelles for delivering various polyphenols, including curcumin.

Favorable disposition of curcumin and doxorubicin was achieved when these drugs were combined in PEG micelles for cervical and hepatic cancer [47]. Few studies have documented a profound toxicity of curcumin-loaded poloxamer nanocarriers towards HeLa [48] and ovarian cancer cells [49]. In addition, poloxamer nanoformulations containing resveratrol and doxorubicin exhibited a synergistic effect on ovarian cancer in mice [50]. A resveratrol–quercetin combination exhibited the same effect in ovarian tumors [51]. Moreover, resveratrol was encapsulated into PEG–polycaprolactone conjugate, and the resulting micelles were surface-modified with apolipoprotein and used for the treatment of glioblastoma [51] and breast cancer [52]. Lastly, some other studies reported epigallocatechin gallate delivery in colon cancer from PEG–polylactic acid [53] and in pancreas cancer from casein micelles [54]. The micelles of various polymers, such as PEG and polycaprolactone, showed an improved anticancer efficacy of the loaded polyphenols, such as quercetin, resveratrol, and curcumin.

### 2.2. Polymer-Based Nanoparticles

High stability, uniform particle size, excellent drug loading efficiency, and controlled release of drug are important characteristics of polymeric nanoparticles [55], which are spherical or irregular shaped, colloidal systems loaded with drugs [56]. A wide range of biocompatible, natural, and synthetic polymers have been utilized as polymeric nanoparticles to deliver anticancer drugs [57,58]. Table 3 illustrates the representative examples of polymers used as nanoparticles for the delivery of polyphenols. Due to their biocompatible and biodegradable features, chitosan and polylactic-*co*-glycolic acid PLGA have been extensively studied for polyphenol delivery [59]. To prevent the uptake of nanoparticles by macrophages, the surface functionalization of nanoparticles can be modified by using polyethylene glycol PEG and its derivatives [60]. The selection of the procedure for the fabrication of polymeric nanoparticles depends on various factors, such as the properties of the employed polymer, drug, and the desired end product to achieve the desired, controllable physicochemical and pharmacological performance in vitro and in vivo. Table 4 also depicts some extensively employed approaches, such as emulsion solvent removal, polymer interaction, and radical polymerization.

Compared with free polyphenols, polyphenol extracts loaded into chitosan, PLGA– polycaprolactone nanoparticles exhibited boosted apoptosis induction and cell internalization, resulting in the enhanced antiproliferative activity in various cell line studies [61,62,63].

Curcumin is a pharmacologically active polyphenol with low water solubility. Therefore, many studies have been conducted to prepare its effective formulations. In this context, an important effort is the development of curcumin-loaded nanoparticles. Therapeutic studies involving various cancer cell lines, including cervical and prostate cells, osteoclasts, and melanocytes [64,65,66,67,68], revealed that these nanoparticles exhibited controlled release of curcumin, resulting in effective passive targeting. It is noteworthy that both free curcumin and curcumin-loaded nanoparticles have the same mechanism of action. In addition, curcumin-loaded nanoparticles have been synthesized by a free radical polymerization method using polyethylene glycol acrylate, *N*-isopropylacrylamide, and *N*-vinyl-2-pyrrolidone for the treatment of pancreatic cancer. These nanoparticles showed insignificant toxicity in mouse [69]. Another study reported the synthesis of curcumin-loaded nanoparticles by an emulsion polymerization method using chitosan and butyl-cyanoacrylate together for the treatment of hepatic cancer [69]. In addition, free curcumin and curcumin nanoparticles were compared in various cell lines, such as colon, prostate, and ovarian. The nanoparticles of curcumin induced cellular uptake and the apoptosis boosting resulting in the ameliorated anticancer activity than its free form [70,71,72]. PLGA nanoparticles containing PEG were fabricated to improve curcumin efficacy against prostate and colon cancer [73,74,75], while curcumin–silk fibroin nanoparticles have been shown to have a potential role in human hepatocellular carcinoma Hep3B, human neuroblastoma Kelly cells, and human bone marrow-derived mesenchymal stem cells hBMSCs [76]. Moreover, curcumin was encapsulated into pH-responsive nanogels to enhance its efficacy against colon cancer [75]. To achieve a synergistic effect, curcumin nanoparticles containing conventional anticancer drugs, such as doxorubicin [77] and 5-fluorouracil [78], have been employed for breast cancer treatment. For the treatment of ovarian cancer, a useful association between curcumin- and cisplatin-loaded nanoparticles has been noted [79]. Furthermore, curcumin combined with gemcitabine in nanoparticles, prepared by free radical polymerization using *N*-isopropylacrylamide, *N*-vinyl-2-pyrrolidone, and acrylic acid, exhibited a synergistic anticancer effect in animal models [80]. Thus, compared to that of free curcumin, curcumin nanoparticles induce cellular uptake, and the apoptosis boosting leads to increased anticancer activity in various cell lines, such as colon, prostate, and ovarian.

Using natural polymers, such as gelatin [81] and a PLGA–PEG combination [82], as well as synthetic polymers, including chitosan–casein–PEG derivatives [82], the synthesis of epigallocatechin gallate nanoparticles with improved stability and in vitro activity against various organs, such as prostate, alimentary canal, breast, and stomach [81,82,83,84], was achieved. Furthermore, epigallocatechin gallate nanoparticles containing doxorubicin were prepared which exhibited a synergistic anticancer effect against Ehrlich ascites cancer [85]. In vivo studies in xenograft mice have also proved the effective stability and activity of epigallocatechin gallate nanoparticles against stomach, prostate, and melanocyte carcinoma [86,87,88]. In addition, epigallocatechin gallate combined with cisplatin in a nanoparticulate formulation was developed as a new synergistic therapy for some invasive cancers [89,90].

Some studies reported the nanoencapsulation of resveratrol into bovine serum albumin [91], gelatin [92], PLGA [93], and PLGA–PEG derivatives [94], revealing an increase in resveratrol activity against cancer of various organs, such as prostate, ovaries, breasts, and lungs [91,92,93,94]. Resveratrol-loaded PLGA–PEG nanoparticles were surface-modified using transferrin for active targeting of glioma cancer cells in vivo [95].

Quercetin and 5-fluorouracil were co-encapsulated into chitosan, and the resulting nanoparticles showed a synergistic effect against pancreatic cells in vitro [96]. Another synergistic study described the promising potential of quercetin–tamoxifen loaded into PLGA nanoparticles for the treatment of breast cancer in model mice [97]. Lastly, a four-component system was formulated using poly-butyl cyanoacrylate, α-tocopherol, and PEG for the delivery of hyaluronic acid into liver cancerous cells in vitro [98]. The preparation of nanoparticles loaded with epigallocatechin gallate, resveratrol, quercetin, and 5-fluorouracil with improved stability and in vitro activity against various organs, such as stomach, prostate, ovaries, alimentary canal, and breast, can be achieved using various natural polymers, such as gelatin, PEG, and PLGA, alone and in combination with synthetic polymers, such as chitosan and casein.

### 2.3. Polymer-Based Conjugates

An important class of the emerging systems for the treatment of cancer is polymer-based conjugates, which consist of a drug molecule and a hydrophilic polymeric macromolecule covalently bonded to each other. In recent years, tremendous research has been conducted to explore new and functional therapeutic conjugates. Like nanoparticles, polymeric conjugates are also high molecular weight systems that affect a drug’s pharmacokinetics, toxicity, and efficacy [99].

Polymer–drug conjugate–a water-soluble system is composed of a drug-associating unit, another unit for linking an active targeting molecule, such as monoclonal antibody, and a portion for linking an element useful for the modulation of physicochemical features [100]. The therapeutic potential of polymeric conjugates is profoundly improved by using antioxidant polymers, which can be acquired either by the conjugation of polyphenol monomers with macromolecules or the polymerization of monomer units of polyphenols. High molecular weight antioxidants can be prepared by three different approaches, namely, enzymatic catalysis, condensation, and radical grafting [101].

Enzymatic catalysis refers to the catalyst-mediated chemical reaction between non-toxic reagents in milder reaction conditions of pH, temperature, and pressure, resulting in the synthesis of distinct structures having controlled chemical properties [102]. In general, a peroxidase or a tyrosinase is used as the catalyst in a coupling reaction.

In condensation reactions, the functional groups of an antioxidant molecule and a polymeric chain react with each other, producing well-defined products with specific mechanical and physical features. As a result of these reactions, the mechanical properties of the product are similar to those of the parent materials. Esterification and acetylation are two important examples of condensation reactions. Generally, these reactions take place in several steps.

Lastly, the radical grafting approach involves free radical coupling between the polyphenol unit and the polymeric moiety in the presence of mild reaction conditions [103], resulting in the synthesis of a characteristic product that retains chemical features of the parent polyphenols.

Polyphenol-loaded polymeric conjugates for the treatment of cancer are summarized in Table 5. For the treatment of pancreatic cancer, a curcumin–gemcitabine combination was loaded with PEG conjugates through a condensation reaction in the presence of carbodiimide [104]. Also, PEG conjugates containing just curcumin have also been prepared for prostate [105] and glioma cancer [106]. Through the same conjugation technique, synergistic cytotoxicity was achieved with resveratrol–bicalutamide–PEG conjugates in breast and cervical cancer cells [107] and quercetin–paclitaxel–carboxymethyl chitosan conjugates in hepatic cancer cells [108]. Another study reported the synthesis of curcumin–dithiopropionic acid copolymer, followed by conjugation with PEG [109]. PEG hydrogels containing triphosgene–curcumin conjugates showed an increased effect against proliferation in breast cancer cells [110].

Compared with the free forms of the tested polyphenols, the anticancer activity of PEG–catechin amides against breast cancer was synergistically increased in the presence of bortezomib [111]. Therapeutic synergism was also observed when hyaluronic acid–epigallocatechin gallate amides containing granzyme B were tested against colon cancer [112]. The therapeutic analysis of catechin–dextran conjugates showed the increased efficacy of catechin in pancreatic cancer cells [113] and in a neuroblastoma model animal [114]. Other studies showed an increase in the anticancer activity of quercetin-loaded polymethacrylic acid conjugates towards cervical cancer [115] and gallic acid-loaded gelatin conjugates towards cervical cancer [116]. All these conjugates were prepared by a free radical approach. For the treatment of hepatic, pancreatic, prostate, glioma, and breast cancer, curcumin, resveratrol, and quercetin in combination with standard anticancer agents, such as paclitaxel, gemcitabine, or bortezomib, have been successfully loaded to polymeric conjugates.

### 2.4. Carbon-Based Nanostructures and Nanohybrids

A class of nano-sized materials, known as carbon nanostructures, is extensively being investigated for its therapeutic applications [117]. The representative examples of this interesting group of compounds are graphene and carbon nanotubes because of their good permeability, cheap availability, excellent physicochemical features, and large surface area for the likely interaction with bioactive compounds [118,119].

**Figure 1:**
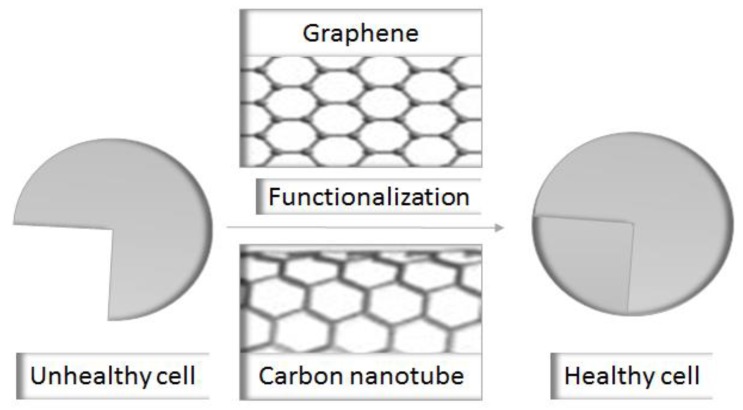
A schematic representation showing the ameliorated effect of functionalization on the cytocompatibility of graphene and carbon nanotubes.

Graphene is a bidimensional honeycomb-like structure, consisting of a layer of six sp^2^ carbon atoms [120]. These bodies undergo cell internalization through endocytosis or active processes [121]. Graphene oxide, an oxidative product of graphene, is an efficient drug delivery vehicle, because it contains numerous functionalities, such as carboxylic and hydroxyl groups Figure 1 [122].

Carbon nanotubes are obtained by the condensation of benzene rings having a composition of sp^2^ carbons, prepared as tube-like structures with a single layer single-walled carbon nanotubes or multiple layers multiple-walled carbon nanotubes [123]. Carbon nanotubes have a strong affinity with different proteins and undergo spiraling movement, thus they are efficiently uptaken by cells, revealing their promising membrane permeability [124].

Graphene oxide and carbon nanotubes are suitable drug delivery vehicles due to their quick physiological distribution, accumulation in various organs, including liver, lungs, kidney, and stomach, and excretion through bile and urine [125,126,127]. In addition, graphene oxide is a biocompatible and cytotoxic substance [128,129]. However, carbon nanotubes could be toxic and produce inflammation, necrosis, fibrosis, and granuloma due to their reducing potential: this feature of carbon nanotubes may hinder their use in drug delivery [129].

These toxicity problems can be eliminated by combining these materials with biocompatible, water-soluble compounds, especially polymers, generating carbon nanohybrids [130].

Numerous studies have reported the successful application of graphene oxide and carbon nanotubes in drug delivery for cancer therapy [131]; however, only a few studies describe their role in the delivery of polyphenols. For instance, a promising modality describes the polyphenol-induced reduction of graphene oxide, resulting in the bond formation between polyphenols and graphene oxide [132]. In this regard, tea polyphenol extract nanohybrids exhibited an improved antiproliferative action in colon cancer cells [133]. Similarly, the proliferation was profoundly inhibited by resveratrol nanohybrids in ovarian cancer cells [134].

On the other hand, pristine carbon nanotubes have been used in some studies for the delivery of polyphenols [135]. Owing to their toxic features, carbon nanotubes have been made biologically compatible by coating with suitable polymers, including gelatin Table 6. In this context, multiple-walled carbon nanotubes were combined with polycaprolactone, resulting in the formation of nanohybrids. These nanohybrids loaded with tea polyphenol exhibited a promising therapeutic effect towards hepatic and lung cancer [136].

Functional nanohybrids Table 6 have also been prepared by developing covalent bonds between the polyphenol and the polymer through a radical reaction. In this regard, catechin–gelatin conjugate [137,138] and quercetin–methacrylic acid conjugate [139,140] were used as the coating material for multi-walled carbon nanotubes. The obtained nanotherapeutics were found to have enhanced anticancer activity in HeLa cancer cells, compared with the free flavonoids [137,139]. It is remarkable that a synergistic anticancer effect can be achieved by using these flavonoid nanohybrids and radiotherapy together towards neuroblastoma [140] and prostate cancer treatment [138]. All these studies demonstrated that carbon nanotubes and graphene oxide could be successfully utilized for the delivery of the polyphenols, including quercetin and catechins, for the effective treatment of cancer, including hepatic, prostate, and lung cancer.

### 2.5. Magnetic Nanoparticles Manipulation of Nanoparticles Using Magnetic Field

**Figure 2:**
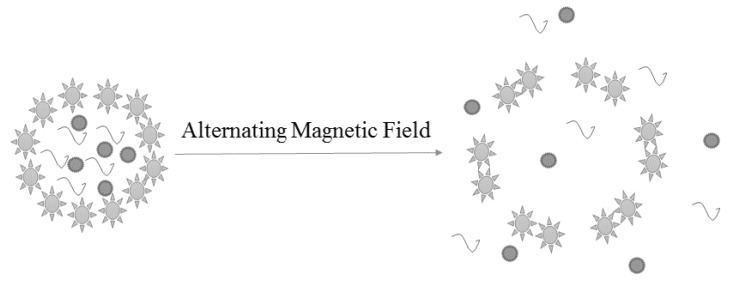
A concept figure showing drug release from magnetic nanoparticles under the effect of alternating magnetic field.

The nanoparticles modulated by a magnetic field, termed magnetic nanoparticles, are extensively studied drug delivery vehicles for the treatment of inflammation, cancer, and other chronic diseases [141,142]. In addition to remote actuation, an alternate magnetic field with high radiofrequency can be applied for the heating of nanoparticles Figure 2 to augment the microenvironment temperature and enhance the probability of synergism.

In recent years, several studies Table 7 have reported the application of magnetic nanoparticle as a vehicle for the delivery of polyphenols for the treatment of tumors. It has been reported that curcumin conjugates possess profound cytotoxicity in Caco-2 cells, glioma [143], and breast cells [144]. Another study described the improved pharmacokinetics and cytotoxicity of curcumin–poloxamer nanoparticles, compared with curcumin alone [145]. Furthermore, magnetic nanoparticles coated with catechin–dextran conjugate exhibited an excellent anticancer activity towards pancreatic cancer [146]. A similar therapeutic outcome was observed when colon cancer cells were treated with epigallocatechin gallate–dextran conjugate [147]. The in vitro treatment of SMMC-7721 tumor cells with quercetin-loaded nickel nanoparticles exhibited synergism between the therapeutic effect and the permeability-enhancing effect of quercetin and nickel nanoparticles, respectively [148]. The nanocarriers for the delivery of polyphenols are studied in vivo to a limited extent, likely due to the fact that these nanoparticles, like any nano-sized drug delivery system, circulate for a short time in the blood as well as exhibit non-specific features. A representative study [147] reporting in vivo experiments on green tea-coated magnetic nanocrystals described their promising transport and uptake properties, suggesting their potential use in therapeutics and multimodal imaging.

## 3. Conclusions

In spite of extensive research struggles, the limitations to achieving effective cancer therapy are still unresolved. Similarly, natural products, including polyphenols, have been known for their anticancer effects for a long time, but their clinical use is still a dream. The above discussion reveals that the exclusive use of polyphenols as cancer therapy is inadequate for translation into therapeutic protocol; rather, due to the substantial synergism observed in study models, polyphenols can be suggested in combination with standard therapeutic modalities. Moreover, it is encouraging that a wide range of safe and effective polymeric nanoparticulate systems are available for the delivery of multiple compounds. Thus, polyphenols could be recommended for clinical use in the future.

## Figures and Tables

**Table 1:** Polyphenol-loaded polymersomes for the treatment of cancer.

No.	Components of Nanoparticles	Method of Preparation	Polyphenol + Synergistic Agent	Type of Cancer In Vitro Model/In Vivo Model Promisingly Ttreated with the Fabricated Nanotherapeutic Formulation	References
1	Polyvinyl pyrrolidone–PEG	Emulsion evaporation	Plant polyphenols	Glioblastoma DBTRG-05MG	[28]
2	Keratin	Solvent evaporation	Curcumin	Cervical cancer HeLa	[29]
3	Gelatin	Solvent evaporation	Curcumin	Lung cancer H1299	[30]
4	PEG–Oleic acid	Thin layer evaporation	Curcumin	Brain cancer U87MG	[42]

PEG: polyethylene glycol.

**Table 2:** Polyphenol-loaded polymeric micelles for the treatment of cancer.

No.	Components of Nanoparticles	Method of Preparation	Polyphenol + Synergistic Agent	Type of Cancer In Vitro Model/In Vivo Model Promisingly Treated with the Fabricated Nanotherapeutic Formulation	References
1	Gelatin–Dextran	Self-assembly-Genipin-Crosslinking	Plant polyphenols	Breast cancer MCF-7	[26]
2	Gelatin–Dextran	Self-assembly-Genipin-Crosslinking	Curcumin	Cervical cancer HeLa Healthy mice	[27]
3	Casein	Self-assembly	Curcumin	Cervical cancer HeLa	[32]
4	Zein–PEG	Self-assembly	Curcumin	Ovarian cancer NCI Healthy mice	[33]
5	Chitosan–Stearic acid	Self-assembly	Curcumin	Colon cancer Primary Xenograft mice	[34]
6	PEG–Polyanhydride esters	Solvent evaporation	Curcumin	Cervical cancer HeLa	[35]
7	PEG–Polylactic acid	Solvent evaporation	Curcumin + Doxorubicin	Breast cancer MCF-7 Xenograft mice	[36]
8	PEG–Polycaprolactone	Thin-layer evaporation	Curcumin	Ovarian cancer A2780t	[38]
9	PEG–Polycaprolactone	Thin-layer evaporation	Curcumin	Breast cancer MDA-MB-436	[37]
10	PEG–Polycaprolactone	Self-assembly	Curcumin	Breast cancer 4T1–4T1 Xenograft mice	[40]
11	PEG–Polycaprolactone	Thin-layer evaporation	Curcumin	Cervical cancer HeLa Xenograft mice	[39]
12	PEG–Polycaprolactone	Thin-layer evaporation	Curcumin	Colon HT-29	[39]
13	PEG–Polycaprolactone	Thin-layer evaporation	Curcumin + Doxorubicin	Lung cancer LL/2 Xenograft mice	[41]
14	Linoleic acid-PEG-Polycaprolactone	Self-assembly	Curcumin	Cervical cancer HeLa Healthy mice	[43]
15	Linoleic acid-PEG-Polycaprolactone	Self-assembly	Curcumin	Lung A549	[43]
16	PEG -Palmitic acid	Self-assembly	Curcumin	Cervical cancer HeLa	[32]
17	PEG2000-DSPE	Thin-layer evaporation	Curcumin + Paclitaxel	Ovarian cancer SK-OV-3TR	[45]
18	PEG2000-DSPE	Thin-layer evaporation	Curcumin + Paclitaxel	Ovarian cancer NCI SK-OV-3TR Xenograft mice	[46]
19	PEG2000-DSPE	Thin-layer evaporation	Curcumin + Doxorubicin	Colon cancer HCT-116 Xenograft mice	[44]
20	PEG- Doxorubicin	Self-assembly	Curcumin + Doxorubicin	Cervical cancer HeLa HepG2 Xenograft mice	[47]
21	PEG-Doxorubicin	Self-assembly	Curcumin + Doxorubicin	Hepatic HepG2	[47]
22	Poloxamers F127 F68	Thin-layer evaporation	Curcumin	Cervical cancer HeLa	[48]
23	Poloxamers-PEG-Succinate	Solvent evaporation	Curcumin	Ovarian cancer NCI	[49]
24	Poloxamers F127	Thin-layer evaporation	Resveratrol, Curcumin + Doxorubicin	Ovarian cancer SKOV-3 Healthy mice	[50]
25	Poloxamers F127	Thin-layer evaporation	Resveratrol, Quercetin + Doxorubicin	Ovarian cancer SKOV-3 Healthy mice	[31]
26	Apolipoprotein-E3	recombinant DNA	Resveratrol	Glioblastoma A-172	[51]
27	Polycaprolactone-PEG-Succinate	Thin-layer evaporation	Resveratrol	Breast cancer MCF-7	[52]
28	Casein	Self-assembly	Epigallocatechin gallate	Colon cancer HT-29	[53]
29	Polylactic acid-PEG	Thin-layer evaporation	Epigallocatechin gallate	Pancreatic cancer MiaPaca-2	[54]

**Note:** PEG2000-DSPE—1,2-distearoyl-*sn*-glycero-3-phosphoethanolamine-*N*-[methoxypolyethylene glycol-2000].

**Table 3:** Polyphenol-loaded polymeric nanoparticles for the treatment of cancer in vitro.

No.	Components of Nanoparticles	Method of Preparation	Polyphenol + Synergistic Agent	Type of Cancer In Vitro Model In Vivo Model Promisingly Treated with the Fabricated Nanotherapeutic Formulation	References
1	Polylactic-co-glycolic acid PLGA–PEG	Emulsion solvent evaporation	Pomgranade polyphenols	Breast cancer MCF-7, Hs578T	[61]
2	Chitosan	Ionic gelation	Tea polyphenols	Hepatic cancer Hep G2	[62]
3	Polycaprolactone	EXP	Plant polyphenols	Gastric cancer MNK28	[63]
4	Alginate–Chitosan–Poloxamers F127	Ionic gelation	Curcumin	Cervical cancer HeLa	[64]
5	Fibrinogen	CaCl_2_ Crosslinking	Curcumin	Prostate cancer PC3	[65]
6	PLGA	Emulsion solvent evaporation	Curcumin	Breast cancer MCF-7	[65]
7	PLGA	Emulsion solvent evaporation	Curcumin	Osteosarcoma U2OS	[66]
8	Chitin	Emulsion solvent evaporation	Curcumin	Melanoma A375	[67]
9	Peptide	Ionic gelation	Curcumin	Medulloblastoma DAOY	[68]
10	*N*-Isopropylacrylamide-*N*-vinyl-2-pyrrolidone-Polyethylene glycol acrylate	Self-assembly	Curcumin	Pancreatic cancer Capan-1, MiaPaCa2, PL-5, PL-8, Su86.86, BxPC-3, PANC-1, E3LZ10.7 Healthy mice	[69]
11	PLGA–PEG	Nanoprecipitation	Curcumin	Colon cancer HT-29 Healthy mice	[70]
12	PLGA	Nanoprecipitation	Curcumin	Ovarian cancer A2780, A2780CP	[71]
13	Cellulose	Nanoprecipitation	Curcumin	Prostate cancer C4-2, PC-3, LNCaP, DU-145	[72]
14	PLGA	Nanoprecipitation	Curcumin	Prostate cancer DU-145, PC-3 Xenograft mice	[73]
15	Human serum albumin	Emulsion solvent evaporation	Curcumin	Colon cancer HCT116 HCT116 Xenograft mice	[74]
16	Human serum albumin	Emulsion solvent evaporation	Curcumin	Pancreatic cancer MiaPaCa2	[74]
17	Gelatin–Polyacryl-amidoglycolic acid	Emulsion polymerization	Curcumin	Colon cancer HCT-116	[75]
18	Silk fibroin	Physical adsorption and coprecipitation	Curcumin	Human hepatocellular carcinoma Hep3B, human neuroblastoma Kelly cells, Human bone marrow-derived mesenchymal stem cells hBMSCs	[76]
19	Chitosan–Polybutyl cyanoacrylate	Emulsion polymerization	Curcumin + Doxorubicin	Breast cancer MCF-7	[77]
20	PLGA	Emulsion solvent evaporation	Curcumin + 5-fluorouracil	Breast cancer MCF-7	[78]
21	PLGA	Nanoprecipitation	Curcumin + Cisplatin	Ovarian cancer A2780CP	[79]
22	PLGA	Nanoprecipitation	Curcumin + Cisplatin	Breast cancer MDA-MB-231	[79]
23	*N*-Isopropylacryl-amide-*N*-vinyl-2-pyrrolidone–Acrylic acid	Radical polymerization	Curcumin + Gemcitabine	Pancreatic cancer Pa03C Xenograft mice	[80]

**Table 4:** Polyphenol-loaded polymeric nanoparticles for the treatment of cancer in vitro.

No.	Components of Nanoparticles	Method of Preparation	Polyphenol + Synergistic Agent	Type of Cancer In Vitro Model In Vivo Model Promisingly Treated with the Fabricated Nanotherapeutic Formulation	References
1	Gelatin–Polyelectrolyte	Layer-by-layer	Epigallocatechin gallate	Breast cancer MBA-MD-231	[81]
2	PLGA–PEG	Nanoprecipitation	Epigallocatechin gallate	Prostate cancer LNCaP	[82]
3	Casein-phospho-peptide–Chitosan	Genipin-Crosslinking	Epigallocatechin gallate	Hepatic cancer HepG2	[83]
4	Casein-phospho-peptide–Chitosan	Genipin-Crosslinking	Epigallocatechin gallate	Gastric cancer BGC823	[83]
5	Casein-phospho-peptide–Chitosan	Genipin-Crosslinking	Epigallocatechin gallate	Colon cancer Caco-2	[84]
6	Hyaluronic acid	Self-assembly	Epigallocatechin gallate + Doxorubicin	Cancer of the external auditory canal	[85]
8	Chitosan	Ionic gelation	Epigallocatechin gallate	Prostate cancer 22R_1 Xenograft mice	[87]
7	Chitosan	Ionic gelation	Epigallocatechin gallate	Melanoma Mel928 Mel928 Xenograft mice	[88]
9	Chitosan–Gelatin–PEG	Ionic gelation	Epigallocatechin gallate	Gastric cancer Luc MKN45 Xenograft mice	[88]
10	PLGA	Nanoprecipitation	Epigallocatechin gallate + Cisplatin	Lung cancer A549	[89]
11	PLGA	Nanoprecipitation	Epigallocatechin gallate + Cisplatin	Cervical cancer HeLA	[89]
12	PLGA	Nanoprecipitation	Theaflavin	Leukemia THP-1	[89]
13	PLGA	Solvent evaporation	Epigallocatechin gallate + Cisplatin	Lung cancer A549 Ehrlich ascites carcinoma Xenograft mice	[90]
14	PLGA	Solvent evaporation	Epigallocatechin gallate	Cervical cancer HeLA	[90]
15	PLGA	Solvent evaporation	Theaflavin	Leukemia THP-1	[90]
16	PLGA	Solvent evaporation	Theaflavin	Cancer of the external auditory canal	[90]
17	PLGA–PEG	Nanoprecipitation	Resveratrol	Prostate cancer DU-145, LNCaP	[91]
18	Bovine serum albumin	Nanoprecipitation	Resveratrol	Lung cancer NCI-H460	[92]
19	Bovine serum albumin	Nanoprecipitation	Resveratrol	Ovarian cancer SKOV3	[93]
20	PLGA	Emulsion method	Resveratrol	Breast cancer MCF-7	[94]
21	Maleimide–PEG–Polylactic acid	Self-assembly	Resveratrol	Glioblastoma CT26, U87 CT26 Xenograft mice	[95]
22	Chitosan	Ionic gelation	Quercetin + 5-fluorouracil	Pancreas cancer MiaPaCa2	[96]
23	PLGA	Emulsion solvent evaporation	Quercetin + Tamoxifen	Breast cancer MCF-7 Xenograft mice	[97]
24	PLGA	Emulsion solvent evaporation	Quercetin + Tamoxifen	Colon cancer Caco2	[97]
25	Hyaluronic acid–Polybutyl cyanoacrylate–a-Tocopheryl–PEG–Succinate	Radical polymerization	Morin hydrate	Lung cancer A549 S180 Xenograft mice	[98]
26	Hyaluronic acid–Polybutyl cyanoacrylate –Tocopheryl–PEG–Succinate	Radical polymerization	Morin hydrate	Hepatic cancer L02	[98]

**Table 5:** Polyphenol-loaded polymeric conjugates for the treatment of cancer.

No.	Components of Nanoparticles	Method of Preparation	Polyphenol + Synergistic Agent	Type of Cancer In Vitro Model In Vivo Model Promisingly Treated with the Fabricated Nanotherapeutic Formulation	References
1	PEG	Condensation method	Curcumin	Glioma C6	[106]
2	PEG	Condensation method	Curcumin	Prostate cancer PC-3	[105]
3	PEG	Condensation method	Curcumin + Gemcitabine	Pancreatic cancer MiaPaCa2, PANC-1, BxPC-3, AsPC-1	[104]
4	PEG	Condensation method	Resveratrol + Bicalutamide	Cervical cancer HeLa	[107]
5	PEG	Condensation method	Resveratrol + Bicalutamide	Breast cancer MCF-7	[107]
6	Carboxymethyl chitosan	Condensation method	Quercetin + Paclitaxel	Hepatic cancer HepG2 HepG2 Xenograft mice	[108]
7	PEG	Condensation method	Curcumin	Cervical cancer HeLa, Breast cancer EMT6 EMT6 Xenograft mice	[109]
8	PEG–Desaminotyrosyl-tyrosine ethyl ester	Condensation method	Curcumin	Breast cancer MDA-MB-231	[110]
9	PEG	Condensation method	Catechin + Bortezomib	Breast cancer MDA-MB-231	[111]
10	Hyaluronic acid–Polyethyleneimine	Condensation method	Epigallocatechin gallate + Granzyme B	Colon cancer HCT-116	[112]
11	Dextran	Free radical grafting	Catechin	Pancreatic cancer MiaPaca-2, PL45	[113]
12	Dextran	Free radical grafting	Catechin	Neuroblastoma IMR-32, IMR-32-CisRes, BE2-C Xenograft mice	[114]
13	Dextran	Enzyme laccase catalysis	Catechin	Neuroblastoma IMR-32	[114]
14	Polymethacrylic acid	Free radical grafting	Quercetin	Cervical cancer HeLa	[115]
15	Gelatin	Free radical grafting	Gallic acid	Prostate cancer DU-145, PC-3	[116]
16	Gelatin	Free radical grafting	Gallic acid	Renal cancer A498	[116]

**Table 6:** Polyphenol-loaded carbon-based nanohybrids for the treatment of cancer.

No.	Components of Nanoparticles	Method of Preparation	Polyphenol + Synergistic Agent	Type of Cancer In Vitro Model/In Vivo Model Promisingly Treated with the Fabricated Nanotherapeutic Formulation	References
1	Graphene oxide	Reduction method	Tea polyphenols	Colon cancer HT29, SW48	[133]
2	Graphene oxide	Reduction method	Resveratrol	Ovarian cancer A2780	[134]
3	Polycapro-lactone–MWNT	Electrospinning	Tea polyphenols	Lung cancer A549	[136]
4	Polycapro-lactone–MWNT	Electrospinning	Tea polyphenols	Hepatic HepG2	[136]
5	Gelatin–MWNT	Coating	Catechin + Radiotherapy	Prostate cancer DY-145, PC-3, LNCap	[138]
6	Gelatin–MWNT	Coating	Catechin	Cervical cancer HeLa	[139]
7	Polymeth-acrylic acid–MWNT	Radical coupling	Quercetin	Cervical cancer HeLa	[137]
8	Polymeth-acrylic acid–MWNT	Radical coupling	Quercetin + Cisplatin	Neuroblastoma IMR-32	[140]

**Note:** MWNT—Multiple-walled carbon nanotubes.

**Table 7:** Polyphenol-loaded magnetic nanoparticles for the treatment of cancer.

No.	Components of Nanoparticles	Method of Preparation	Polyphenol + Synergistic Agent	Type of Cancer In Vitro Model/In Vivo Model Promisingly Treated with the Fabricated Nanotherapeutic Formulation	References
1	Hyaluronic acid–Iron	Layer-by-layer	Curcumin	Colon cancer Caco-2	[143]
2	Polyvinyl pyrrolidone–Iron	Layer-by-layer	Curcumin	Glioma C6	[143]
3	Iron–Poloxamers F127	Nanopre-cipitation	Curcumin	Pancreatic cancer HPAF-II, Panc-1/Xenograft mice	[145]
	Iron–Dextran	Solvation method	Catechin	Pancreatic cancer MIA Paca2	[146]
4	Iron	Reduction process	Epigallocatechin gallate	Colon cancer CT-26/Xenograft mice	[147]
5	Nickel	Electro-chemical deposition	Quercetin	Hepatic cancer SMMC-7721	[148]
